# Heat protection behaviour in the UK: results of an online survey after the 2013 heatwave

**DOI:** 10.1186/s12889-015-2181-8

**Published:** 2015-09-10

**Authors:** Swarna Khare, Shakoor Hajat, Sari Kovats, Carmen E. Lefevre, Wändi Bruine de Bruin, Suraje Dessai, Angie Bone

**Affiliations:** Department of Social and Environmental Health Research, London School of Hygiene and Tropical Medicine, 15-17 Tavistock Place, London, WC1H 9SH UK; Centre for Decision Research, Leeds University Business School, Maurice Keyworth Building, The University of Leeds, Leeds, LS2 9JT UK; Sustainability Research Institute and ESRC Centre for Climate Change Economics and Policy, School of Earth and Environment, University of Leeds, Leeds, LS2 9JT UK; Extreme Events and Health Protection, Centre for Radiation, Chemicals and Environmental Hazards, Public Health England, Wellington House 133-155 Waterloo Road, London, SE1 8UG UK

## Abstract

**Background:**

The Heatwave Plan for England provides guidance for personal and home protection measures during heatwaves. Although studies in the USA, Australia and Europe have surveyed heat-related behaviours during heatwaves, few have been conducted in the UK. This study assesses personal and housing (at-home) behaviour and housing characteristics of the UK population during the 2013 heatwave.

**Methods:**

This paper analyses data from 1497 respondents of an online survey on heat protection measures and behaviour. Participants were asked questions about their behaviour during the 2013 heatwave, the characteristics of their current housing as well as about any negative health outcomes experienced due to the hot weather. We used multinomial logit regression to analyse personal and home heat protection behaviour and logistic regression to analyse characteristics of participants’ current home (installed air conditioner, curtains etc.). We stratified the outcomes by age, sex, ethnicity, income, education and regional location.

**Results:**

In 2013, for all heat-related illness (except tiredness), a higher proportion of those in the younger age groups reported symptoms compared with those in the older age groups. Women, higher income groups and those with higher education levels were found to be more likely to report always/often taking personal heat protective measures. The elderly were less likely to take some personal and home protective measures but were more likely to live in insulated homes and open windows at night to keep their home cool.

**Conclusion:**

Our study has found a high level of awareness of the actions to take during heatwaves in the UK, and has identified important demographic indicators of sections of the UK population that might benefit from additional or more targeted information. The health agencies should attempt to provide better information about heatwaves to those vulnerable (elderly, those at risk living in London, low income earners) or identify any barriers that might be preventing them from undertaking protective behaviour.

## Background

July 2013 was the third warmest in the national (central England) temperature record going back to 1910, with a mean temperature of 17 °C, behind 2006 (17.8 °C) and 1983 (17.3 °C). The heatwave of 2013 was notable for its duration rather than intensity, with prolonged high temperatures for 19 consecutive days (from the 6^th^ of July to the 24^th^ of July). A maximum of 28 °C was recorded at one or more locations on each of those 19 days [1].

The Heatwave Plan for England sets out various levels of heatwave alert and has been published annually since 2004 by the Department of Health in England (and by Public Health England since 2013), following the 2003 heatwave when more than 2000 deaths were attributed to the heatwave in England and Wales [2]. Heatwaves in England are declared when the threshold maximum day temperature (average across all regions is 30 °C) and a minimum night temperature (average across all regions is 15 °C) are exceeded for at least two consecutive days [3]. In July 2013, there were five level 2 (‘heatwave is forecast’) and nine level 3 alerts (‘heatwave action’), with at least a level 2 being experienced in all areas of England, except North East England. There were no heatwave alerts in Scotland, Wales or Northern Ireland as these countries of the UK do not run this service [4].

The Heatwave Plan aims to raise awareness and prevent the major avoidable effects on health during periods of severe heat in England. It recommends a range of protective measures to the public, health and other services such as drinking fluids and staying out of the heat, keeping the environment cool, and looking out for others in vulnerable groups using sunscreen, wearing protective clothing and sunglasses and to avoid the sun around 4 h of midday and to seek shade whenever possible [5].

The Heatwave Plan for England classifies older people, especially those over 75 years old and female, babies and children, and homeless people as being at higher risk of health effects during a heatwave. A qualitative study in London and Norwich, UK found that most elderly people did not consider themselves to be at risk but did follow some “common sense” guidelines during heatwaves [6]. Internationally, a few surveys have been conducted (in Portugal, France, USA and Canada), as found by a systematic review [7], to assess the general public response to heatwave warnings and to assess change in practices among the general public during a heatwave. It was found that although awareness of heat events is widespread, very few of those potentially vulnerable were changing their behaviour accordingly. Similarly, it was found from a survey across four North American cities, that awareness of heat warnings was almost universal but only half of all respondents mentioned that they changed their behaviour [8]. A later study in Adelaide, Australia, examined participants’ knowledge about heatwaves and also their adaptive behaviours during heatwaves and although they found that over 80 % of their sample had good adaptive behaviour [9], the same is not true in other countries as highlighted above.

Apart from personal heat protection behaviour, housing/dwelling characteristics have also been found to be important. Opening windows at night, using mechanical fans and air conditioners have been found to be significant protective factors against mortality due to heat [10–13]. Very few studies have, however, looked at home protection behaviour in the UK during a heatwave but have instead focused on recommendations about home characteristics to deal with overheating during heatwaves in the UK [14, 15].

This paper uses data collected from a survey conducted by Research Now after the 2013 heatwave to analyse a number of heat protection recommendations (as outlined in the Heatwave Plan for England) undertaken by the general public in the UK. To fill in an important gap in the evidence base, we assess personal behaviour, housing behaviour and housing characteristics reported by the survey participants during the heatwave in 2013. We also analyse the proportion of respondents who reported experiencing various heat related health outcomes such as dehydration, heat stroke, sunburn etc. during the heatwave. Our aim is to investigate which groups within the population report undertaking heat protective measures and their demographic profile.

## Methods

This paper analyses data from a national online survey on heat protection measures and behaviour, which was conducted in October 2013 by a survey research company, Research Now, after the high temperatures experienced throughout the UK in July 2013. Using two rounds of targeted email invitations, a sample of 1497 adult UK participants (18 years and over) was recruited. The survey was closed when the targeted number of participants was met, thus the response rate of 13.59 % partially reflects speed of responding. The sample was representative of the UK population in terms of age and sex, although the age groups over 65 years were deliberately oversampled at a rate of 2:1, because they are most at-risk of heat-related health effects. A total of 35 % of respondents did not complete the survey after having started it, likely because of its length. Those who did not complete the survey were significantly younger (43.8 years vs. 52.4 years; *p* < .001) and more likely to be female (56 % vs. 50 %; *p* = .005) than the final sample. Sample characteristics and their comparison to the UK population are presented in Table 1 [16]. Our sample was less ethnically diverse and higher educated, as compared to the overall population (all *p* < .001). Additionally, our sample was markedly older than the general population, due to our strategy to oversample older adults.

**Table 1:** Demographic information of sample on selected variables

Variable	Statistic Total N (%)	UK population (%)
Age:	Mean: 54.37, SD = 18.82	Mean: 45.00, SD = 27.27
18-25	160 (10.7%)	10.6%
26-60	743 (49.7%)	46.3%
61-75	405 (27.1%)	14.5%
76+	187 (12.5%)	7.3%
Sex:		
Female	754 (50.5 %)	50.8 %
Male	735 (49.3 %)	49.2 %
Ethnicity:		
White	1407 (94.4 %)	86.0 %
Non White	83 (5.6 %)	14.0 %
Highest level of Education:		
GCSE/O level/vocational level 2, Level 1 and below or no qualification	492 (32.9 %)	51.2 %
A level/vocational level 3	304 (20.3 %)	12.3 %
Higher Education	660 (44.1 %)	27.2 %
Other, including foreign	38 (2.5 %)	5.7 %
Regional location		
East and Midlands	533 (36.6 %)	25.3 %
London	172 (11.8 %)	12.9 %
North	266 (18.3 %)	23.6 %
South	343 (23.6 %)	22.0 %
Scotland	68 (4.7 %)	8.4 %
Wales	58 (4.0 %)	4.8 %
Northern Ireland	16 (1.1 %)	2.9 %
Annual pre-tax household income:		
< £15,000	288 (20.1 %)	20.2 %
£15,000 - £29,999	528 (36.9 %)	32.2 %
£30,000 - £49,999	387 (27.0 %)	24.2 %
> £50,000	229 (16.0 %)	23.4 %

Participants were asked questions relating to their experience of the 2013 heatwave as well as general questions about heat protection measures and overall health [17]. Initially, different participants were asked to think of different temperatures at the start of the survey (participants were asked to think of the most unpleasant temperature, most unpleasant highest temperature, highest temperature they could recall from the heatwave of 2013). However, an analysis of responses by the different temperature groups found not enough significant differences between groups and therefore we pooled all participants’ responses in this analysis. This paper analyses the responses received for questions on heat protection measures and behaviour, heat related health outcomes and heat protection measures in the respondents’ home.

We divided the responses into four categories:

Firstly, participants’ experience of heat illness during the heatwave of 2013 was assessed by asking “During the heatwave in the summer 2013, did you experience the following outcomes as a result of heat?” Participants then indicated ‘yes’ or ‘no’ for each of the following: dehydration, heat stroke, headaches, dizziness, nausea or vomiting, confusion, aggression, convulsions, loss of consciousness, tiredness, sun burn, and missed work.

Participants were then asked which personal heat protection behaviours they had engaged in during the summer of 2013 including: keeping out of the sun between 11.00 am and 3.00 pm; walking in the shade; applying sunscreen; avoiding extreme physical exertion (such as exercise, running, or playing sports); having plenty of cold drinks; avoiding excess alcohol; keeping an eye on isolated, elderly or ill people and on babies and children to make sure they were able to keep cool; and using an electric fan. For each option participants answered always/often, rarely/occasionally or never.

Participants were also asked about the heat protection measures in their home including: keeping windows closed during the day; opening windows at night; and closing curtains of windows that received morning or afternoon sun. For each option participants answered always/often, rarely/occasionally or never.

Participants were then asked about the heat protection characteristics of their current homes which included having shutters, light and dark curtains, portable and installed air conditioners and loft/wall insulation, to which participants responded with either a yes or no.

Heat illness outcomes were not analysed by means of regression analysis but results have been presented as proportion of participants (by age group) who did report experiencing these. The reason for doing this has been laid out in the “Discussion”.

*Multinomial logit regression models* were fitted to assess personal heat protection behaviour and participants’ home protection measures (if the behaviour of interest was exhibited “always/often”, “rarely/sometimes” or “never”) and a *logistic regression model* was fitted to assess heat protection characteristics of participants’ current homes (whether a specific home characteristic was installed in the participant’s home or not). The independent variables for each of the three models were age group, sex, ethnicity, regional location, education level and income.

## Results

Table 1 shows the demographic and socio-economic characteristics of the survey participants. There were approximately equal numbers of male and female participants. It can be seen that the largest percentage of participants were in the 26–60 years age group. Additionally there were no males in the youngest and oldest age groups in Northern Ireland. Eight participants had not specified a gender and 61 males and 55 females did not specify where they lived.

### Heat illness outcomes

**Fig. 1:**
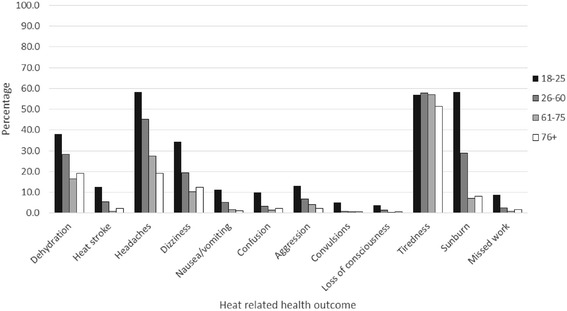
Percentage of respondents who reported a heat related health symptom by age group during the 2013 heatwave

Figure 1 shows the percentage of respondents who reported a heat illness symptom in each age group. From left to right, the black bar represents proportion of 18–25 year olds who reported experiencing each symptom, followed by those aged 26–60, 61–75 and finally the white bar represents those older than 75 years. More respondents in the youngest age group (18–25) reported experiencing each outcome (except tiredness) than those in the oldest age groups. Approximately 60 % of all 18–25 year olds reported suffering from a headache and sunburn in the heatwave of 2013 compared to only 20 % and 8 % respectively of all those older than 75 years. Over half of the respondents in each age group had reported feeling tired during the heatwave of summer 2013.

### Heat protection behaviour in 2013 – personal measures

Only the comparison between those who always/often undertook personal heat protection measures relative to those who never did (base category) is discussed below and shown in Table 2 because few significant differences were found between those who rarely/occasionally undertook personal heat protection measures relative to those who never did. Where variables were in fact significant in this group they were also significant in the always/often group. Due to this and to avoid a lengthy repeated discussion, we only report results comparing the always/often group relative to the base category.

**Table 2:** Multivariate relationships between heat protection behaviour and significant risk factors

Personal heat protection measures	Number and percentage of respondents who always/often undertook the personal heat protection measure	Significant risk factors (α = 0.05)	Relative risk ratio [95 % CI]
Always keeping out of the sun between 1100 and 1500	*N = 783 % = 52.4*	*Age group (base: 18–25)*	
		26-60	1.0 [0.4, 2.5]
		61-75	2.7 [1.0, 7.7]
		76+	1.4 [0.5, 4.2]
		*Sex (base: male)*	
		Female	2.2 [1.3, 3.8]*
		*Ethnicity (base: Non White)*	
		White	1.9 [0.8, 4.8]
		*Location (base: South of England)*	
		East of England and the Midlands	0.7 [0.3, 1.4]
		London	0.5 [0.2, 1.4]
		North	0.6 [0.3, 1.5]
		Scotland	0.1 [0.0, 0.3]*
		Wales	1.2 [0.2, 5.7]
		Northern Ireland	0.1 [0.0, 0.6]*
		*Education level (base: higher level/university qualification)*	
		A-Levels/vocational level 3	0.7 [0.4, 1.4]
		GCSE O Levels/vocational level 2	1.1 [0.6, 2.0]
		Other/ unknown qualification	0.4 [1.0, 1.4]
		*Income (base: <£15,000 gross yearly)*	
		£15,000 - £29,999 gross yearly	0.8 [0.4, 1.8]
		£30,000 - £49,999 gross yearly	1.1 [0.5, 2.4]
		> £50,000 gross yearly	1.0 [0.4, 2.5]
Always walk in the shade	*N = 893 % = 59.9*	*Sex (base: male)*	
		Female	2.9 [1.5, 5.5]
		*Location (base: South of England)*	
		Scotland	0.2 [0.0, 0.9]
		Northern Ireland	0.1 [0.0, 0.5]
Always apply sunscreen	*N = 887 % = 59.4*	*Age group (base: 18–25)*	
		61-75	0.3 [0.1, 0.7]
		76+	0.2 [0.1, 0.7]
		*Sex (base: male)*	
		Female	4.2 [2.7, 6.6]
		*Ethnicity (base: Non White)*	
		White	4.1 [1.8, 9.4]
		*Income (base: <£15,000 gross yearly)*	
		£15,000 - £29,999 gross yearly	1.9 [1.1, 3.1]
		£30,000 - £49,999 gross yearly	3.0 [1.6, 5.5]
Always avoid excess physical activity (heavy exercising, sports etc.)	*N = 1041 % = 69.6*	*Sex (base: male)*	
		Female	3.0 [1.3, 6.9]
Always have plenty of cold drinks	*N = 1248 % = 83.6*	*None*	
Always avoid excess alcohol	*N = 968 % = 65.1*	*Sex (base: male)*	
		Female	2.9 [1.5, 5.6]
		*Education level (base: higher level/university qualification)*	
		Other/ unknown qualification	0.2 [0.1, 0.8]
Always keep an eye on ill or elderly people	*N = 596 % = 39.9*	*Sex (base: male)*	
		Female	1.6 [1.1, 2.3]
		*Education level (base: higher level/university qualification)*	
		O-level/vocational level 2	1.7 [1.1, 2.6]
Always keep an eye on babies	*N = 683 % = 45.8*	*None*	

*significant risk factor at α = 0.05

**Fig. 2:**
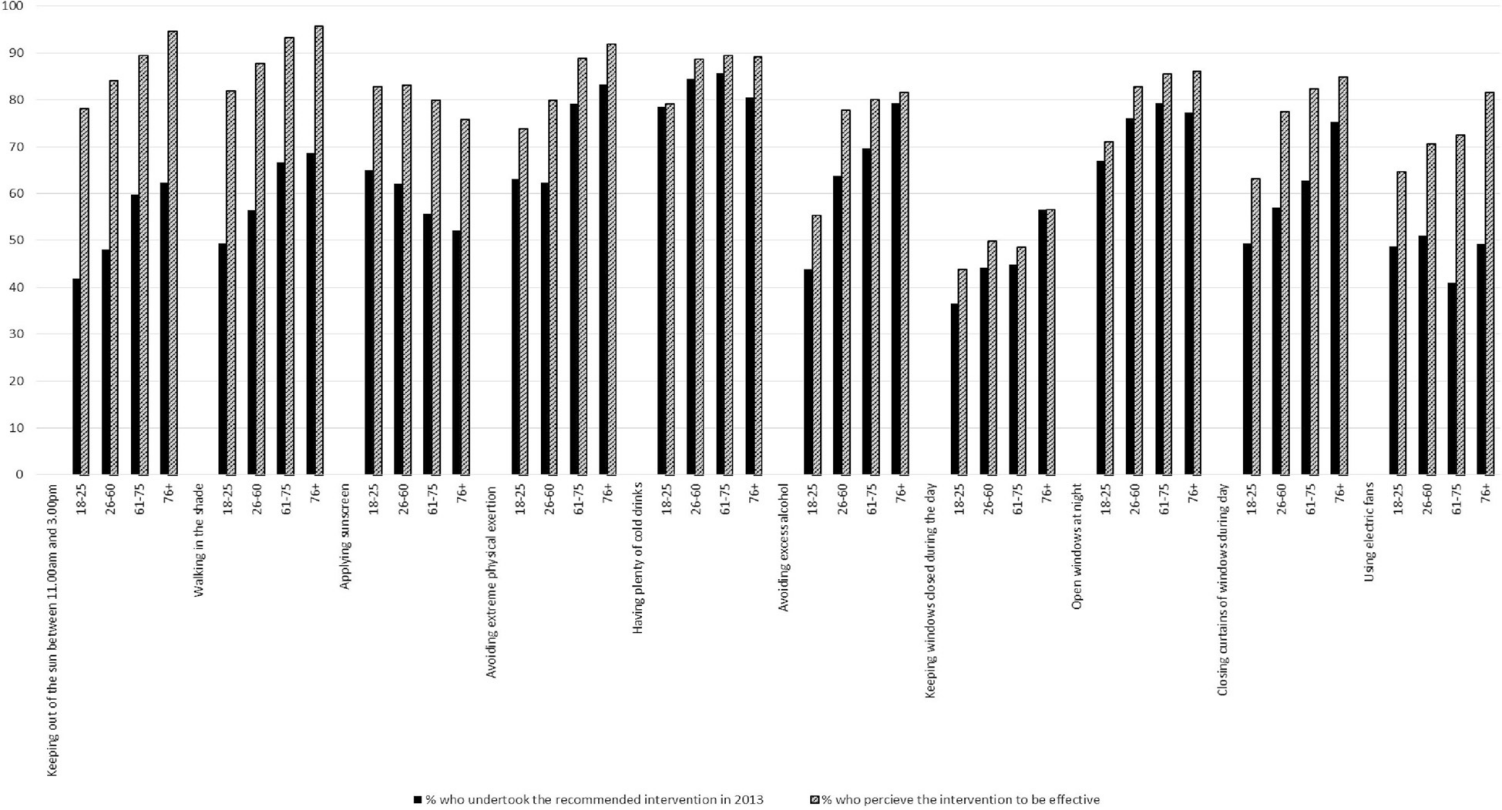
General perception of effectiveness of heat measures actually taken during the 2013 heatwave: by age group

Figure 2 shows that all age groups found the recommended heat protection measures (both home and personal) to be generally effective/very effective but they did not always/often use them during the heatwave of 2013. For example although over 75 % of participants in all age groups said that they believed that keeping out of the sun between 1100 and 1500 h is an effective measure against heat, only approximately half of all participants always/often avoided the sun in these hours. Similarly more participants in all age groups believed that applying sunscreen is an effective protection measure than those who always or often applied sunscreen. Similar results were observed for all other heat protection measures.

The results are shown in Table 2. The full model with all the independent variables used in the multinomial logit regression model is shown for the first outcome (always/often keeping out of the sun between 1100 and 1500 h). For all subsequent outcomes only the significant variables are shown.

In the fitted multinomial regression model gender was found to be a significant predictor for most of the personal heat protection behaviours undertaken. During the heatwave of 2013, women were at least twice as likely as men to always avoid the sun between 1100 and 1500 h (RR = 2.2; 95 % CI: 1.3-3.8), walk in the shade (RR = 2.9; 95 % CI: 1.5-5.5), avoid physically exhausting activities (heavy exercise, sports) (RR = 3.0; 95 % CI: 1.3-6.9) and avoid excess alcohol (RR = 2.9; 95 % CI: 1.5-5.6). They were also 4 times as likely as men to always apply sunscreen (RR = 4.2; 95 % CI: 2.7-6.6) and were significantly more likely than men to always keep an eye on ill or elderly people to make sure they were able to keep cool during the hot weather (RR = 1.6; 95 % CI: 1.1-2.3).

Income was also a significant factor with higher income earners being more likely to apply sunscreen (£15,000-£29,999, RR = 1.9, 95 % CI: 1.1-3.1; £30,000-£49,999, RR = 3.0; 95 % CI: 1.6-5.5).

Those educated up to GCSE/O Levels were more likely to keep an eye on ill or elderly people than those with a higher level qualification (RR = 1.7; 95 % CI: 1.1-2.6). However those with “other” qualifications (where the level is not known and including foreign qualifications) were 80 % less likely to avoid excess alcohol compared to those with higher educational qualifications (RR = 0.2; 95 % CI: 0.1-0.8).

Respondents from Scotland and Northern Ireland were at least 80 % less likely to always avoid the sun between 1100 and 1500 h (Scotland: RR = 0.1, 95%CI:0.0-0.3; NI: RR = 0.1, 95 % CI: 0.0-0.6) or walk in the shade (Scotland: RR = 0.2, 95%CI:0.0-0.9; NI: RR = 0.1, 95 % CI: 0.0-0.5) compared to respondents from the South of England.

White respondents were four times as likely as non-white respondents to apply sunscreen whereas respondents older than 61 years of age were less likely to apply sunscreen compared to respondents aged between 18–25 years (61–75: RR = 0.3, 95 % CI: 0.1-0.7; older than 75: RR = 0.2, 95 % CI: 0.1-0.7).

### Home protection measures

The results are shown in Table 3. The full model with all the independent variables used in the multinomial logit regression model is shown for the first outcome (keep windows that receive the afternoon sun closed during the day). For all subsequent outcomes only the significant variables are shown.

**Table 3:** Multivariate relationships between home protection measures and significant risk factors

Home heat protection measures	Number and percentage of respondents who always/often undertook the home heat protection measure	Significant risk factors (α = 0.05)	Relative risk ratio [95 % CI]
Keep windows that receive the afternoon sun closed during the day	*N = 670 % =45.1*	*Age group (base: 18–25)*	
		26-60	1.0 [0.5, 2.0]
		61-75	0.7 [0.4, 1.5]
		76+	1.1 [0.5, 2.5]
		*Sex (base: male)*	
		Female	1.5 [1.0, 2.1]*
		*Ethnicity (base: Non White)*	
		White	0.9 [0.4, 1.9]
		*Location (base: South of England)*	
		East of England and the Midlands	1.1 [0.7, 1.8]
		London	0.7 [0.4, 1.2]
		North	0.9 [0.5, 1.6]
		Scotland	0.4 [0.2, 0.8]*
		Wales	0.6 [0.2, 1.3]
		Northern Ireland	0.4 [0.1, 2.3]
		*Education level (base: higher level/university qualification)*	
		A-Levels/vocational level 3	0.9 [0.5, 1.4]
		GCSE O Levels/vocational level 2	0.9 [0.6, 1.4]
		Other/ unknown qualification	0.6 [0.2, 1.9]
		*Income (base: <£15,000 gross yearly)*	
		£15,000 - £29,999 gross yearly	1.8 [1.1, 2.9]*
		£30,000 - £49,999 gross yearly	1.2 [0.7, 2.0]
		> £50,000 gross yearly	1.5 [0.8, 2.7]
Open windows at night	*N = 1139 % = 76.2*	*Age group (base: 18–25)*	
		26-60	2.6 [1.1, 6.2]
		61-75	3.5 [1.3, 9.2]
		76+	3.9 [1.2, 12.4]
		*Education level (base: higher level/university qualification)*	
		O-level/vocational level 2	0.5 [0.2, 0.9]
		*Income (base: <£15,000 gross yearly)*	
		£30,000 - £49,999 gross yearly	2.6 [1.0, 6.8]
Close curtain of windows that receive the afternoon sun	*N = 895 % = 60.0*	*Location (base: South of England)*	
		London	0.5 [0.2, 1.0]
		North	0.5 [0.3, 1.0]
Always use electric fans	*N = 710 % = 47.8*	*Age group (base: 18–25)*	
		61-75	0.5 [0.3, 0.9]
		*Location (base: South of England)*	
		East of England and Midlands	1.7 [1.1, 2.5]
		Scotland	0.3 [0.2, 0.7]
		*Education level (base: higher level/university qualification)*	
		A-level/vocational level 3	2.8 [1.8, 4.5]
		O-level/vocational level 2	1.9 [1.3, 2.7]
		*Income (base: <£15,000 gross yearly)*	
		£15,000 - £29,999 gross yearly	1.8 [1.2, 2.7]

*Significant variables at α = 0.05

It was found that respondents from Scotland were 60 % less likely to keep windows that received the afternoon sun closed during the day (RR = 0.4, 95 % CI: 0.2-0.8). Women and higher income earners were 50 % (RR = 1.5, 95 % CI: 1.0-2.1) and 80 % (RR = 1.8, 95 % CI: 1.1-2.9) respectively more likely to do this.

It was also found that the older age groups were significantly more likely to open their windows at night compared to those in the youngest age group (26–60: RR = 2.6, 95 % CI: 1.1-6.2; 61–75: RR = 3.5, 95 % CI: 1.3-9.2; older than 75: RR = 3.9, 95 % CI: 1.2-12.4). Additionally, higher income earners were more than 2.5 times as likely as those earning less than £15,000 gross yearly to open their windows at night (RR = 2.6; 95 % CI: 1.0-6.8) whereas those respondents educated only up to GCSE O-Levels were 50 % less likely to do this compared to those who had higher education/university level qualifications (RR = 0.5; 95 % CI: 0.2-0.9).

Respondents from London and the North of England were both 50 % less likely to close curtain of windows that receive the afternoon sun (London: RR = 0.5, 95 % CI: 0.2-1.0; North of England: RR = 0.5, 95 % CI: 0.3-1.0).

Finally, during the 2013 heatwave, those aged 61–75 years were 50 % less likely to use electric fans as a cooling measure for their home compared to 18–25 years olds (RR = 0.5, 95 % CI: 0.3-0.9). Those from Scotland were also 70 % less likely to use electric fans than those in the South of England (RR = 0.3, 95%CI: 0.2-0.7). On the other hand, respondents from the East of England and the Midlands were 70 % more likely to use electric fans than those from the South of England (RR = 1.7, 95%CI: 1.1-2.5). Those educated up to A-levels (RR = 2.8; 95 % CI: 1.8-4.5) or up to GCSE O Levels (RR = 1.9; 95 % CI: 1.3-2.7) were more likely to use electric fans to keep cool than those with higher university level educational qualifications during last year’s heatwave. Higher income earners were three times as likely to use electric fans (£15,000-£29,999, RR = 2.8; 95 % CI: 1.2-2.7) as those earning less than £15,000 gross annual income, during the heatwave of 2013.

### Home characteristics

The results are shown in Table 4. The full model with all the independent variables used in the multinomial logit regression model is shown for the first characteristic (installed air conditioner). For all subsequent characteristics only the significant variables are shown.

**Table 4:** Multivariate relationships between home characteristics and significant risk factors

Home characteristics	Number and percentage whose home had a specific characteristic	Significant factors (α = 0.05)	Relative risk ratio [95 % CI]
Installed air conditioner	*N=47, %=3.2*	*Age group (base: 18–25)*	
		26-60	0.4 [0.2, 0.9]*
		61-75	0.2 [0.1, 0.6]*
		76+	0.5 [0.2, 1.6]
		*Sex (base: male)*	
		Female	0.8 [0.4, 1.6]
		*Ethnicity (base: Non White)*	
		White	0.3 [0.1, 0.8]*
		*Location (base: South of England)*	
		East of England and the Midlands	1.5 [0.6, 3.6]
		London	1.5 [0.5, 4.6]
		North	2.0 [0.7, 5.4]
		Scotland	0.0 [0.0, 0.0]
		Wales	1.2 [0.2, 6.1]
		Northern Ireland	0.0 [0.0, 0.0]
		*Education level (base: higher level/university qualification)*	
		A-Levels/vocational level 3	1.0 [0.5, 2.3]
		GCSE O Levels/vocational level 2	0.9 [0.4, 1.9]
		Other/ unknown qualification	0.9 [0.1, 7.4]
		*Income (base: <£15,000 gross yearly)*	
		£15,000 - £29,999 gross yearly	1.7 [0.7, 4.1]
		£30,000 - £49,999 gross yearly	1.1 [0.4, 3.1]
		> £50,000 gross yearly	0.9 [0.3, 3.0]
Dark curtains	*N = 911, % = 61.7*	*None*	
Portable air conditioner	*N = 265, % = 18.1*	*Age group (base: 18–25)*	
		26-60	0.5 [0.3, 0.8]
		61-75	0.5 [0.3, 0.9]
Shutters	*N = 172, % = 11.6*	*Age group (base: 18–25)*	
		26-60	0.4 [0.2, 0.7]
		61-75	0.3 [0.2, 0.5]
		76+	0.5 [0.2, 0.9]
		*Ethnicity (base: Non White)*	
		White	0.4 [0.2, 0.6]
		*Location (base: South of England)*	
		London	2.1 [1.2, 3.8]
Loft/wall insulation	*N = 1144, % = 77.2*	*Age group (base: 18–25)*	
		26-60	1.7 [1.1, 2.6]
		61-75	3.4 [2.1, 5.5]
		76+	3.5 [1.9, 6.2]
		*Location (base: South of England)*	
		London	0.4 [0.3, 0.7]
		*Income (base: <£15,000 gross yearly)*	
		£15,000 - £29,999 gross yearly	1.5 [1.0, 2.1]
		£30,000 - £49,999 gross yearly	1.5 [1.0, 2.2]
		>£50,000 gross yearly	1.6 [1.0, 2.5]
Light curtains	*N = 1085, % = 73.4*	*Age group (base: 18–25)*	
		61-75	2.0 [1.3, 3.1]
		*Location (base: South of England)*	
		London	0.6 [0.4, 1.0]
		*Education level (base: higher level/university qualification)*	
		O-level/vocational level 2	0.7 [0.5, 0.9]

*Significant variables at α = 0.05

Most participants reported having curtains and wall insulation in their home. Only 3 % of the sample had reported having installed air conditioners which is consistent with the national average [18]. It was found that age groups 26–60 years and 61–75 years were at least 50 % less likely to use shutters (26–60: RR = 0.4, 95 % CI: 0.2-0.7; 61–75: RR = 0.3, 95 % CI: 0.2-0.5; older than 75: RR = 0.5, 95 % CI: 0.2-0.9) or air conditioners (either installed (26–60: RR = 0.4, 95 % CI: 0.2-0.9; 61–75: RR = 0.2, 95 % CI: 0.1-0.6) or portable (26–60: RR = 0.5, 95 % CI: 0.3-0.8; 61–75: RR = 0.5, 95 % CI: 0.3-0.9)) in their home compared to those aged 18–25 years.

However 26–60 year olds were 70 % and 61 years and older were 240 % more likely to have loft/wall insulation in their home compared to those in the youngest age group (26–60: RR = 1.7, 95 % CI: 1.1-2.6; 61–75: RR = 3.4, 95 % CI: 2.1-5.5; older than 75: RR = 3.5, 95 % CI: 1.9-6.2). 61–75 year olds were also twice as likely to use light curtains in their home as those in the youngest age group (RR = 2.0; 95 % CI: 1.3-3.1).

White respondents were also less likely than non-white respondents to use shutters (RR = 0.4; 95 % CI: 0.2-0.6) or install air conditioners (RR = 0.3; 95 % CI: 0.1-0.8) in their home.

Respondents from London were twice as likely to use shutters in their home compared to those from the South of England (RR = 2.1; 95 % CI: 2.1-3.8). However, they were less likely to make use of light curtains (RR = 0.6; 95 % CI: 0.4-1.0) or have loft/wall insulation (RR = 0.4; 95 % CI: 0.3-0.7) in their home, compared to those from the South of England. Similarly those educated up to GCSE O-Levels were 30 % less likely to use light curtains to protect their home from heat (RR = 0.7; 95 % CI: 0.5-0.9).

Finally, higher income led to progressively higher odds of having a loft/wall insulation in the home compared to those earning less than £15,000 gross annually. (£15,000-£29,999: RR = 1.5, 95 % CI: 1.0-2.1; £30,000-£49,999: RR = 1.5, 95 % CI: 1.0-2.2; >£50,000: RR = 1.6, 95 % CI: 1.0-2.5).

## Discussion

### Heat illness outcomes

We began by looking at heat illness outcomes as reported by the participants in different age groups. A higher percentage of those in younger age groups reported experiencing each outcome especially sunburn and headaches. Of particular interest is the result for heat stroke which over 10 % of the 18–25 year-olds reported having experienced in the heatwave of 2013. This is unlikely to be true heatstroke as this is a serious condition with a high fatality rate. As an indication of the national prevalence of heat illness, the NHS syndromic surveillance report issued by Public Health England, calls made to NHS Direct for heat/sunstroke were less than 0.5 % of the total number of calls made [19]. Overall, there is strong reason to suspect that prevalence indicated by self-reported health outcomes may be biased. Higher rates of reporting in the younger age group may also be related to the group being more likely to engage in “risky” behaviours such as increased alcohol consumption during hot weather. The self-reported health outcomes are not a valid measure of clinical heat illness, and the results needs to be interpreted with caution.

Thus, instead of examining factors relating to the reporting of these health outcomes, this study aims to examine the socio-demographic factors that are significantly associated with reporting the adoption of heat protection measures. The heat protection measures are divided into personal measures and home measures.

### Personal and home heat protection behaviour

Clear gender differences were found between men and women in reporting personal and home protection measures. Women were more likely to report taking up both personal and home protection recommendations than men. While gender in itself may not be predictor of suffering adversely from hot weather, women tend to live longer than men, so that there are proportionately more women than men in older age-groups nationally [20] as well as in our sample. Older people in general are more likely to experience limiting long-term illness or disability, and are at potentially greater risk of being socially isolated [21]. Results reported by EuroHEAT found that elderly females in Mediterranean cities were at a significantly higher risk of mortality than males [22] and a study by Vaneckova et.al. [23] found similar results for two regions in Sydney, Australia. As such, our results indicate a better uptake of heat protection recommendations amongst women compared to men which, given the evidence of women being more vulnerable, is a positive observation. In addition, there is some evidence that women are known to be more to likely to seek information on health risks and undertake adaptive behaviours than men [24, 25].

Similarly high income earners reported a higher uptake of both personal and home protection measures compared to the lowest income group. This is consistent with findings from a US study, which reported that low income earners, although more aware of and concerned about climate change, faced more barriers to adapting their behaviour to a heat spell because of lack of resources and not knowing how to change their behaviour as well as due to lack of time [26]. The same study also found that higher education levels were a significant predictor of behaviour change and we found the same to be true for the UK. However, our study found that the highly educated were less likely to report the use of electric fans in their homes in the UK which may be due to them wanting to reduce personal contributions to climate change, as found in the study conducted in the USA.

There were also age related differences in uptake of recommended home and personal protection measures with older respondents less likely to report the use of sunscreens, electric fans and air conditioners than those in the youngest age group. Studies in the UK have found that the elderly do not perceive themselves to be at risk or vulnerable to extreme heat and strongly felt that babies and the disabled were at a higher risk; in addition, these studies found that serious health effects of heat were poorly understood [6, 27].

Finally, participants in Scotland and Northern Ireland were less likely to engage in both personal and home protection behaviour compared to those in South of England. This may be due to the fact that maximum temperatures in these countries over summer months tend to keep under 20 °C on average (Met Office - Weather Averages) [28]. Both countries do not presently have a heatwave plan which might be an additional reason why respondents from these countries were less likely to take precautionary measures. Overall, within England, there were no marked regional differences in personal and home heat protection behaviours, with the exception that participants in London were less likely to take home protection measures. This might only be due to the fact that although Londoners were less likely to use curtains in their home they were found to be twice as likely to use shutters instead. However, a study of vulnerability to mortality due to heat has found that Londoners are at the highest risk compared to other English regions, followed by those in the East of England [29].

### Strength and what our study adds

This is the first national study of attitudes and behaviour of the public in response to heatwaves in the UK. It complements surveys undertaken in other European countries, the United States and Australia. In this way this study has identified important demographic indicators of those sections of the UK population that might face barriers to application of heat protection behaviour even after they receive all information about recommended measures to take during hot weather. For example, there is a need to understand the barriers that might be facing the elderly, those at risk living in London, and low income earners that might prevent them from taking precautions, suggested by the Heatwave Plan, during periods of extreme heat.

Finally, this study benefits from a large sample of 1497 respondents which is a considerably larger sample than most studies and surveys we have considered in this paper.

### Limitations

This being a self-report questionnaire based study there may be limitations regarding response bias with regards to heat related health outcomes as well as heat protection measures reportedly taken. For example the high percentage of reported health outcomes in the 18–25 year age group might be due to factors other than heat or due to reporting of self-diagnosed heat related illness which may not have been brought to the attention of medical professionals at all.

Regional differences may not be accurately represented because of few participants from Scotland, Wales and Northern Ireland. There might also be urban–rural differences in heat protection behaviour which cannot be explored in this study due to lack of very precise locational information.

Finally, the survey was conducted in October 2013, a few months after the summer heatwave of that year. It is possible that due to the time lapse participants were unable to accurately recall the heat measures that they in fact took in the warmer months.

As with all self-reported survey analyses we have had to assume that participants’ answers to survey questions are as close as possible to their actual behaviour.

## Conclusion

Our study found some evidence that the elderly, despite being a vulnerable group, were not considerably more likely to always/often take personal heat protection measures compared to younger age groups, but were more likely to keep their homes cool by using curtains and opening their windows at night. Low income groups and residents in London have been found to be at a high risk of heat related mortality by other studies and government reports [30, 31] but our study indicates that they were less likely to take some protective measures. This may have implications for public health organisations and highlights the importance of timely and effective communication of heat protective strategies to such groups. Additionally there is a strong need to study barriers to uptake of heat protection measures.
